# Differences in Nutritional Intake, Total Body Fat, and BMI Score between Twins

**DOI:** 10.3390/nu14173655

**Published:** 2022-09-04

**Authors:** So Young Kim, Dae Myoung Yoo, Mi Jung Kwon, Ji Hee Kim, Joo-Hee Kim, Woo Jin Bang, Hyo Geun Choi

**Affiliations:** 1 Bundang CHA Medical Center, Department of Otorhinolaryngology-Head and Neck Surgery, CHA University, Seongnam 13488, Korea; 2 Hallym Data Science Laboratory, Hallym University College of Medicine, Anyang 14066, Korea; 3 Department of Pathology, Hallym Sacred Heart Hospital, Hallym University College of Medicine, Anyang 14068, Korea; 4 Department of Neurosurgery, Hallym University College of Medicine, Anyang 14068, Korea; 5 Department of Medicine, Division of Pulmonary, Allergy, and Critical Care Medicine, Hallym Sacred Heart Hospital, Hallym University College of Medicine, Anyang 14068, Korea; 6 Department of Urology, Hallym Sacred Heart Hospital, Hallym University College of Medicine, Anyang 14068, Korea; 7 Department of Otorhinolaryngology-Head and Neck Surgery, Hallym University College of Medicine, Anyang 14068, Korea

**Keywords:** obesity, fat, carbohydrate, twin, cohort studies

## Abstract

The present study aimed to investigate the coincidence of obesity and nutritional intake in monozygotic twins compared to dizygotic twins. The data from the Korean Genome and Epidemiology Study (KoGES) from 2005 through 2014 were analyzed. Participants ≥ 20 years old were enrolled. The 1006 monozygotic twins and 238 dizygotic twins were analyzed for differences in self-reported nutritional intake, total body fat, and body mass index (BMI) using a linear regression model. The estimated values (EV) with 95% confidence intervals (95% CI) of the difference in dietary intake, total body fat, and BMI score were calculated. The monozygotic twin group and the dizygotic twin group showed similar differences in nutritional intake, DEXA fat, and BMI (all *p* > 0.05). The differences in nutritional intake of total calories and carbohydrates were lower in the monozygotic twin group than in the dizygotic twin group (all *p* < 0.05). The differences in total body fat were lower in monozygotic twins than in dizygotic twins (adjusted EV = 2427.86 g, 95% CI = 1777.19–3078.53 and adjusted EV = 1.90%, 95% CI = 1.33–2.46). Monozygotic twins had more similar dietary habits for total calories and carbohydrate intake. Other nutritional factors did not show differential similarity between monozygotic and dizygotic twins. Total body fat was more concordant in monozygotic twins.

## 1. Introduction

Obesity is a common disease whose prevalence is estimated to be approximately 36.9% in men and 38.0% in women in the worldwide adult population [1]. In addition, the population of obesity is increasing worldwide [1]. The high and increasing prevalence of obesity has been one of the major concerns because it has been acknowledged to induce multiple metabolic and cardiovascular complications [2,3]. In the adult population, high body mass index (BMI) was related to about 1.52 times greater risk of cardiometabolic diseases (95% confidence intervals [95% CI] = 1.45–1.58) [4].

Multiple factors can contribute to the occurrence of obesity. Genetic predispositions to obesity have been suggested in twin studies [5]. However, obesity involves multiple predisposing candidate genes that are difficult to dissociate [5]. Monozygotic twin study equalizes background genetic environment between twin pairs. However, many shared and unshared environmental factors need to be considered for their impacts on obesity. In a monozygotic twin study, gut microbiome composition and its metabolites were suggested to be associated with obesity [6]. In that study, the differences in the gut microbiome were presumed to modulate glucose metabolism, which may induce obesity [6]. Because nutritional intake influences the gut microbiome, nutritional intake can influence obesity through modulation of the gut microbiome [7]. In addition, socioeconomic factors, such as education and income levels, also influence obesity [8]. It was reported that high education and income levels were related to a lower risk of obesity (odds ratio (OR) = 0.74, 95% CI = 0.65–0.84 for education level and OR = 0.86, 95% CI = 0.77–0.96 for income level) [8]. Thus, socioeconomic factors should be considered when evaluating the associated factors of obesity.

Previous studies reported a number of genetic factors that result in obesity. In addition, because nutritional intake is a critical factor for obesity, it was questioned whether nutritional intake can be a contributor to obesity as an inherited trait. This study aimed to estimate the inherited portion of obesity compared to the shared environmental factors. To examine these current questions, twin cohorts were analyzed for differences in total body fat, body mass index (BMI), and nutritional intakes. This study is novel in analyzing nutritional intake in twin cohorts and comparing BMI and total body fat. The findings of the current study may enhance the knowledge on the inherited trait for the occurrence of obesity.

## 2. Materials and Methods

### 2.1. Source of Data

The current research was permitted by the ethics committee of Hallym University (2021-03-004). The ethics committee exempted the written informed consent. The Korean Genome and Epidemiology Study (KoGES) from 2005 through 2014 was used [9,10,11,12]. The KoGES Healthy twin Study (HTS) data enrolled the ≥ 20 years old population.

### 2.2. Participants Selection

**Figure 1:**
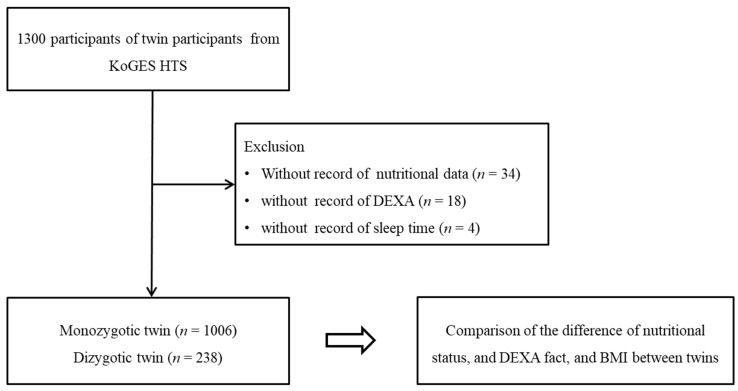
The 1006 of monozygotic twins and 238 of dizygotic twins were compared for the difference of nutritional status, DEXA fat, and body mass index (BMI) between twins.

Among a population of 1300, the participants who did not completed the survey on nutritional intake, dual-energy X-ray absorptiometry (DEXA) exam, and sleep time were excluded. As a result, 1006 monozygotic and 238 dizygotic twin participants were enrolled (Figure 1). The differences on their histories of nutritional intake, DEXA, and BMI score were analyzed between twin pairs.

### 2.3. Survey

The self-reported surveys were conducted for the nutritional intake of total calories (kcal), protein (g), fat (g), carbohydrate (g), calcium (mg), phosphorus (mg), iron (mg), potassium (mg), vitamin A (mg), sodium (mg), vitamin B1 (mg), vitamin B2 (mg), nicotinic acid (mg), vitamin C (mg), zinc (ug), vitamin B6 (mg), folic acid (ug), retinol (ug), carotene (ug), ash (mg), fiber (g), vitamin E (mg), and cholesterol (mg) by trained interviewees using a validated questionnaire [13]. Total body fat was measured using DEXA (Zeus 9.9, g, %). BMI (kg/m^2^) was measured by the automated height-weighing machine in Frankfort Horizontal Plane. The income group was classified based on their household income. Education level, marriage status, and physical activity, walking time, and sitting time were surveyed. Smoking and the frequency of alcohol consumption were self-reported. Sleep time was surveyed with categorized questionnaire of ≤5 h/day, >5 and ≤7 h/day, >7 and ≤9 h/day, and >9 h/day.

### 2.4. Outcome

The absolute difference in dietary intake including fat intake, total body fat, and BMI score between the matched twin participants were estimated.

### 2.5. Statistical Analyses

The categorical variables were compared using chi-square test. The continuous variables were compared using Wilcoxon rank-sum test.

We calculated the estimated values (EV) (absolute difference between monozygotic twins—Absolute difference between dizygotic twins) with 95% CI of the absolute difference of dietary intake, total body fat, and BMI score using a linear regression model.

*p* values < 0.05 were regarded as statistically significant. SPSS v. 24.0 (IBM, Armonk, NY, USA) was used.

## 3. Results

The nutritional intake, DEXA fat, and BMI were not different between the monozygotic and dizygotic twin groups (all *p* > 0.05, Table 1). The distribution of age group and sex and level of physical activity were different between the two groups (both *p* < 0.05). The levels of income and education, marital status, obesity, smoking status, alcohol consumption, and sleep time were not different between the two groups.

The difference in dietary intakes of twin pairs was compared between the monozygotic and dizygotic twin groups (Table 2). The difference in total calories and carbohydrate intakes was lower in the monozygotic group than in the dizygotic group (adjusted EV = 130.95, 95% CI = 22.66–239.24 for total calories; adjusted EV = 19.46, 95% CI = 3.19–35.74 for carbohydrates). All other examined nutritional factors did not demonstrate a significant difference between the two groups for the difference in dietary intakes (all *p* > 0.05).

The difference in total body fat was higher in dizygotic twins than in monozygotic twins (adjusted EV = 2427.86 g, 95% CI = 1777.19–3078.53 and adjusted EV = 1.90%, 95% CI = 1.33–2.46, Table 3). However, the difference in body mass index was not significantly different between monozygotic and dizygotic twins.

## 4. Discussion

The total body fat was more concordant within monozygotic twin pairs than in dizygotic twins. In addition, the concordances in the nutritional intakes for total calories and carbohydrate intakes were higher in monozygotic twins than in dizygotic twins in this study. The current results suggest the inherited portions for the development of obesity and nutritional intake. This study improved the evidence on the inherited contribution of obesity by analyzing monozygotic and dizygotic twins.

The intake of total body fat was more concordant in monozygotic twins than in dizygotic twins in the current study. The metabolic pathways related to body fat storage and adipocyte lipolysis were reported to be related to shared genetic loci [14]. Moreover, a genome-wide association study demonstrated numerous related variants for body fat distribution using UK Biobank data [15]. The inherited traits in adipose metabolism could induce the coincidence of total body fat between monozygotic twin pairs in the current study. It can be presumed that there may be some genetic trait for the occurrence of obesity, which is harbored in monozygotic twins. To support this, a number of previous studies reported genetic factors for the occurrence of obesity [16,17,18].

In addition, nutritional intakes of total calories and carbohydrates were more similar between monozygotic twin pairs than between dizygotic twins in this study. Eating behavior is one of the main determining factors for weight gain [19]. Thus, nutritional intake can be one of the main lifestyle factors for the occurrence of obesity. To support our results, a few twin studies demonstrated the heritability of dietary habits [20,21]. In the young adult population, heritability of the use frequency of food items was calculated to be approximately 40–45% [22]. In the older adult population, the contribution of heritable traits to dietary habits was presumed to be decreased by approximately 15–40% [23]. Because we included the adult population, the heritability of nutritional intake can be diluted for many nutritional factors. However, previous studies also indicated that the heritability of nutritional intake was different according to nutritional factors [20,21]. For example, the heritability of nutritional intake was highest for the percentage of energy from fat, whose heritability was estimated to be approximately 38.7% [20].

However, there was no higher concordance of BMI in monozygotic twin pairs compared to dizygotic twins in the present study. In advance with genome-wide association studies, multiple predisposing genetic factors have been announced for obesity [16]. In addition, epigenetic mechanisms, such as DNA methylation, histone modifications, and posttranscriptional modifications, are related to obesity [16,24]. Therefore, the inherited traits for the occurrence of obesity can be attenuated for other acquired traits, which mitigated the higher co-occurrence of obesity in monozygotic twin pairs than in dizygotic twin pairs in the present study. For instance, shared environmental factors may contribute to the occurrence of obesity [25,26]. In youth, parenting styles, such as emotional support, communication style, and discipline, were associated with BMI [26]. The socioeconomic status shared by family members was suggested to be a risk factor for obesity [27].

This study evaluated the differences in nutritional intake, total body fat, and BMI in monozygotic and dizygotic cohort populations. Total body fat was measured using DEXA, and BMI was calculated based on the measured weight and height. In addition, socioeconomic factors of income level, education, and marital status were considered in the analyses. Furthermore, lifestyle factors of physical activity, smoking, alcohol consumption, and sleep duration were assessed to minimize potential confounding effects from these variables. The KoGES HTS data are generated and regularly monitored by statisticians in the Korean government. However, the cross-sectional study design limited the causality of obesity and nutritional intakes with monozygotic twins in this study. In addition, although validated questionnaires were used to examine nutritional intake [13], there can be recall bias due to the retrospective interview of participants [28,29,30,31,32]. Furthermore, unconcerned possible confounders, such as medication histories related to diet, can influence BMI or total body fat in the current study. Future studies with longitudinal follow-up designs can resolve the current limitations.

## 5. Conclusions

The nutritional intakes of total calories and carbohydrates were more similar in monozygotic twin pairs than in dizygotic twins. Total body fat was more similar between monozygotic twins than between dizygotic twins.

## Figures and Tables

**Table 1:** Demographic factors and nutritional intakes.

Characteristics	Total Participants		
	Monozygotic Twin	Dizygotic Twin	*p*-Value
Age (years old, *n*, %)			0.001 *
20–24	6 (0.6)	0 (0)	
25–29	66 (6.6)	4 (1.7)	
30–34	342 (34)	83 (34.9)	
35–39	234 (23.3)	65 (27.3)	
40–44	132 (13.1)	36 (15.1)	
45–49	128 (12.7)	18 (7.6)	
50–54	80 (8)	22 (9.2)	
55–59	12 (1.2)	10 (4.2)	
60–64	4 (0.4)	0 (0)	
65+	2 (0.2)	0 (0)	
Sex (*n*, %)			0.023 *
Males	368 (36.6)	106 (44.5)	
Females	638 (63.4)	132 (55.5)	
Income (*n*, %)			0.957
<2 million (won)	335 (33.3)	78 (32.8)	
2 to <3 million (won)	266 (26.4)	67 (28.2)	
3 to <4 million (won)	205 (20.4)	48 (20.2)	
≥4 million (won)	200 (19.9)	45 (18.9)	
Education (*n*, %)			0.860
Under high school	117 (11.6)	25 (10.5)	
Graduated from High school	359 (35.7)	87 (36.6)	
Commercial college-Dropped out of college	119 (11.8)	32 (13.4)	
Graduated from High school	411 (40.9)	94 (39.5)	
Marriage (*n*, %)			0.398
Unmarried	233 (23.2)	49 (20.6)	
Married	706 (70.2)	168 (70.6)	
Divorced or others	67 (6.7)	21 (8.8)	
Physical Activity			
Hard (hour/1 week, mean, SD)	3.1 (6.8)	4.7 (9.8)	0.021 *
Moderate (hour/1 week, mean, SD)	5.9 (10.5)	6.2 (10.2)	0.652
Walk (hour/1 week, mean, SD)	6.2 (9.7)	6.6 (10.1)	0.546
Sit (hour/1 week, mean, SD)	40.5 (21.9)	37.8 (20.9)	0.086
Obesity (*n*, %)			0.260
Underweight (BMI < 18.5)	26 (2.6)	5 (2.1)	
Normal (BMI ≥ 18.5 to <23)	484 (48.1)	109 (45.8)	
Overweight (BMI 23 to <25)	212 (21.1)	66 (27.7)	
Obese I (BMI ≥ 25 to <30)	253 (25.1)	52 (21.8)	
Obese II (BMI ≥ 30)	31 (3.1)	6 (2.5)	
Smoking status (*n*, %)			0.112
Nonsmoker	662 (65.8)	141 (59.2)	
Past smoker	102 (10.1)	33 (13.9)	
Current smoker	242 (24.1)	64 (26.9)	
Frequency of drinking alcohol (*n*, %)			0.279
Nondrinker	291 (28.9)	60 (25.2)	
≤1 time per month	228 (22.7)	46 (19.3)	
2–4 times per month	291 (28.9)	79 (33.2)	
≥2 times per week	196 (19.5)	53 (22.3)	
Sleeping hours (*n*, %)			0.314
h ≤ 5	52 (5.2)	16 (6.7)	
5 < h ≤ 7	592 (58.8)	142 (59.7)	
7 < h ≤ 9	336 (33.4)	70 (29.4)	
>9	26 (2.6)	10 (4.2)	
Nutritional intake			
Total calories (kcal, mean, SD)	1851.6 (744.2)	1933.8 (914.1)	0.198
Protein (g, mean, SD)	64.1 (35.8)	66.7 (39.9)	0.340
Fat (g, mean, SD)	35.9 (26.2)	36.6 (32.5)	0.739
Carbohydrate (g, mean, SD)	314.9 (114.4)	330.6 (142.9)	0.116
Calcium (mg, mean, SD)	462.7 (314.8)	463.9 (360.4)	0.961
Phosphorus (mg, mean, SD)	937.1 (474.3)	969 (544.8)	0.364
Iron (mg, mean, SD)	10.6 (6.5)	10.8 (7)	0.582
Potassium (mg, mean, SD)	2362.6 (1336.5)	2411.6 (1507.6)	0.620
Vitamin A (mg, mean, SD)	485.9 (428.8)	478.7 (384.7)	0.813
Sodium (mg, mean, SD)	2547.3 (1628.1)	2710.8 (1674.4)	0.166
Vitamin B1 (mg, mean, SD)	1.1 (0.6)	1.2 (0.8)	0.351
Vitamin B2 (mg, mean, SD)	1 (0.6)	1 (0.7)	0.791
Nicotinic acid (mg, mean, SD)	15.8 (8.7)	16.5 (9.7)	0.289
Vitamin C (mg, mean, SD)	113 (85.2)	110.9 (88.6)	0.740
Zinc (ug, mean, SD)	8.3 (5)	8.7 (4.8)	0.319
Vitamin B6 (mg, mean, SD)	1.6 (0.9)	1.7 (1)	0.288
Folic acid (ug, mean, SD)	221.2 (154.7)	225.3 (168)	0.717
Retinol (ug, mean, SD)	86.3 (74.9)	85.2 (88.9)	0.851
Carotene (ug, mean, SD)	2294.4 (2280.8)	2243.2 (1973.5)	0.750
Ash (mg, mean, SD)	15.3 (10.2)	15.9 (10.2)	0.391
Fiber (g, mean, SD)	5.6 (3.5)	5.7 (3.6)	0.737
Vitamin E (mg, mean, SD)	9 (5.5)	9.3 (7.3)	0.624
Cholesterol (mg, mean, SD)	201.2 (159.6)	198.4 (163.5)	0.808
DEXA fat (g, mean, SD)	16,916 (7404.8)	16,672.2 (7382.5)	0.648
DEXA fat (%, mean, SD)	28.0 (7.6)	26.7 (8.6)	0.622
BMI (kg/m^2^, mean, SD)	23.3 (3.2)	23.4 (3.2)	0.591

* Significance at *p* < 0.05. The difference in dietary intakes of twin pairs was compared between the monozygotic and dizygotic twin groups (Table 2). The difference in total calories and carbohydrate intakes was lower in the monozygotic group than in the dizygotic group (adjusted EV = 130.95, 95% CI = 22.66–239.24 for total calories; adjusted EV = 19.46, 95% CI = 3.19–35.74 for carbohydrates). All other examined nutritional factors did not demonstrate a significant difference between the two groups for the difference in dietary intakes (all *p* > 0.05).

**Table 2:** Estimated values of nutritional intakes.

Difference of Dietary Intake	Monozygotic Twin	Dizygotic Twin	Estimated Values of Absolute Difference between Twin (95% CI)			
	Mean (SD)	Mean (SD)	Crude	*p*-Value	Adjusted †	*p*-Value
Difference of total calories (kcal)	637.6 (600.8)	813.7 (1050.2)	176.06 (75.8 to 276.31)	0.001 *	130.95 (22.66 to 239.24)	0.018
Difference of protein (g)	26.8 (31.6)	33.5 (45.6)	6.73 (1.81 to 11.64)	0.007 *	1.62 (−3.68 to 6.92)	0.548
Difference of fat (g)	19.6 (25)	23.1 (39.2)	3.46 (−0.54 to 7.46)	0.090	0.99 (−3.32 to 5.3)	0.653
Difference of carbohydrate (g)	104.4 (88.2)	132.4 (162.6)	27.96 (12.91 to 43.02)	0.000*	19.46 (3.19 to 35.74)	0.019
Difference of calcium (mg)	256.5 (272.1)	297.4 (383.3)	40.84 (−1.1 to 82.78)	0.056	34 (−11.28 to 79.28)	0.141
Difference of phosphorus (mg)	380.4 (405.6)	465.7 (596.9)	85.34 (21.92 to 148.75)	0.008 *	50.96 (−17.38 to 119.3)	0.144
Difference of iron (mg)	4.8 (5.3)	6 (7.9)	1.2 (0.37 to 2.03)	0.005 *	0.53 (−0.37 to 1.42)	0.249
Difference of potassium (mg)	1129.9 (1162.2)	1280.8 (1640.6)	150.83 (−28.42 to 330.07)	0.099	120.08 (−73.41 to 313.57)	0.224
Difference of vitamin A (mg)	322.7 (419)	327.3 (441.2)	4.58 (−55.29 to 64.45)	0.881	−14.57 (−78.7 to 49.56)	0.656
Difference of sodium (mg)	1412.2 (1363.4)	1486.4 (1565.2)	74.2 (−124.36 to 272.77)	0.464	−163.71 (−377.37 to 49.95)	0.133
Difference of vitamin B1 (mg)	0.5 (0.6)	0.6 (0.9)	0.1 (0.01 to 0.19)	0.032 *	0.02 (−0.08 to 0.12)	0.732
Difference of vitamin B2 (mg)	0.5 (0.5)	0.6 (0.7)	0.07 (−0.01 to 0.15)	0.101	0.05 (−0.04 to 0.13)	0.314
Difference of nicotinic acid (mg)	6.7 (7.7)	8.5 (11)	1.82 (0.62 to 3.01)	0.003 *	0.58 (−0.71 to 1.86)	0.380
Difference of vitamin C (mg)	69.6 (73.7)	81.7 (93.6)	12.14 (1.12 to 23.15)	0.031 *	6.69 (−5.19 to 18.58)	0.269
Difference of zinc (ug)	3.6 (5)	4.4 (5.3)	0.84 (0.12 to 1.55)	0.021 *	0.14 (−0.63 to 0.9)	0.721
Difference of vitamin B6 (mg)	0.7 (0.8)	0.9 (1.1)	0.2 (0.08 to 0.32)	0.001 *	0.05 (−0.08 to 0.18)	0.446
Difference of folic acid (ug)	119.9 (135.7)	141.9 (186.3)	21.99 (1.24 to 42.74)	0.038 *	4.22 (−18.14 to 26.59)	0.711
Difference of retinol (ug)	59.7 (78.5)	66.2 (97.5)	6.5 (−5.17 to 18.17)	0.275	4.86 (−7.59 to 17.31)	0.444
Difference of carotene (ug)	1635 (2286.4)	1685.1 (2286.6)	50.15 (−273.18 to 373.48)	0.761	−82.49 (−429.43 to 264.46)	0.641
Difference of ash (mg)	7.9 (9.8)	9.2 (10.6)	1.24 (−0.16 to 2.65)	0.084	−0.98 (−2.49 to 0.54)	0.207
Difference of fiber (g)	2.8 (2.9)	3.2 (3.9)	0.39 (−0.05 to 0.83)	0.079	0.23 (−0.24 to 0.7)	0.338
Difference of vitamin E (mg)	4.6 (4.5)	6.1 (8.5)	1.53 (0.75 to 2.31)	0.000 *	0.79 (−0.05 to 1.64)	0.066
Difference of cholesterol (mg)	124.2 (146.5)	139.3 (166.8)	15.14 (−6.15 to 36.43)	0.163	−5.13 (−27.85 to 17.59)	0.018

* Significance at *p* < 0.05; † Adjusted for age, sex, income, BMI, education, marriage status, physical activity, smoking habit, frequency of drinking alcohol, and sleep time.

**Table 3:** Estimated values of total body fat and body mass index.

Difference of Clinical Examination	Monozygotic Twin	Dizygotic Twin	Estimated Values Of Absolute Difference Between Twin (95% CI)			
	Mean (SD)	Mean (SD)	Crude	*p*-Value	Adjusted †	*p*-Value
Difference of total body fat (g)	3303.7 (3839.5)	5821.2 (6950)	2517.54 (1866.68 to 3168.40)	<0.001 *	2427.86 (1777.19 to 3078.53)	<0.001 *
Difference of total body fat (%)	3.7 (3.5)	5.7 (5.6)	1.98 (1.42 to 2.54)	<0.001 *	1.90 (1.33 to 2.46)	<0.001 *
Difference of body mass index (kg/m^2^)	23.3 (3.2)	23.4 (3.2)	0.12 (−0.33 to 0.57)	0.591	−0.08 (−0.50 to 0.34)	0.709

* Significance at *p* < 0.05; † Adjusted for age, sex, income, education, marriage status, physical activity, smoking habit, frequency of drinking alcohol, and sleep time.

## Data Availability

Restrictions apply to the availability of these data. Data were obtained from the Korean Genome and Epidemiology Study (KoGES) and are available at https://www.nih.go.kr/contents.es?mid=a50401010100#1 (accessed on 1 January 2022).
